# Abnormal Chest Computed Tomography Findings among Admitted Symptomatic COVID-19 Patients in a Tertiary Care Centre: A Descriptive Cross-sectional Study

**DOI:** 10.31729/jnma.7529

**Published:** 2022-07-31

**Authors:** Sujit Pant, Bina Basnet, Sujata Panta, Neeraj Basanta Tulachan, Kalpana Rai, Mukunda Singh Shrestha

**Affiliations:** 1 Department of Radiology, Nepalese Army Institute of Health Sciences, Syanobharyang, Kathmandu, Nepal

**Keywords:** *COVID-19*, *Nepal*, *pneumonia*, *prevalence*

## Abstract

**Introduction:**

COVID-19 has emerged as a pandemic and has varied clinical presentation. Computed Tomography scans of the chest play an important role in evaluating the lung parenchymal changes and aids in better planning the management of COVID-19 patients. The purpose of this study was to find the prevalence of abnormal chest computed tomography findings among admitted symptomatic COVID-19 patients in a tertiary care centre.

**Methods:**

This descriptive cross-sectional study was conducted from 25 October 2020 to January 2021 in a tertiary care hospital. Ethical approval was taken from the Institutional Review Committee (Registration number: 348). Convenience sampling method was used. Chest computed tomography findings of the admitted symptomatic COVID-19 patients were evaluated for abnormal findings. Point estimate and 95% Confidence Interval were calculated.

**Results:**

Among 153 patients, abnormal chest computed tomography findings were seen in 147 (96.07%) (92.99-99.15, 95% Confidence Interval). The findings of ground-glass opacities with consolidations were seen in 78 (53.06%) patients.

**Conclusions:**

The prevalence of abnormal chest findings among symptomatic COVID-19 patients in our study was similar to the studies done in other countries in similar settings. Majority of the symptomatic COVID-19 patients showed abnormal chest computed tomography scan findings in the form of ground glass opacities and consolidations.

## INTRODUCTION

After worldwide spread of Coronavirus disease 2019 (COVID-19), World Health Organization declared it to be a pandemic.^1-3^ In Nepal, the first case was reported on 24 January 2020 and cases has been increasing since then.^4-5^ Clinical presentation of COVID-19 ranges from symptomatic to critically ill.^6-8^

Reverse transcription polymerase chain reaction (RT-PCR) is considered as the gold standard for diagnosis of COVID-19.^9,10^ But long turn-around-time and chances of false negative report can delay the diagnosis.^2,4^ On the other hand, chest computed tomography (CT) can show suggestive abnormalities before the actual clinical symptoms starts. Chest CT scan therefore is increasingly recognized for early diagnosis and assessment of disease.^9,11,12^ Imaging helps in evaluation of the lung involvement, assessing the severity, planning the management and early detection of the complications.^7,13^

The aim of this study was to find out the prevalence of abnormal chest CT findings in admitted symptomatic COVID-19 patients in a tertiary care centre.

## METHODS

This descriptive cross-sectional study was conducted in Shree Birendra Hospital, Chhauni, Kathmandu, Nepal which was a designated COVID-19 hospital. All symptomatic PCR positive COVID-19 patients admitted in COVID ward or ICU from 25 October 2020 to January 2021 were included in this study. The ethical approval was taken from the Institutional Review Committee (Registration number: 348). Consent was obtained from individual patients, and those who were not able to give the consent, it was obtained from their first-degree relatives. To maintain the privacy of the participants, data was stored in a password protected electronic format. All RT-PCR positive COVID patients admitted with symptoms of COVID-19 such as fever, sore throat, cough, myalgia, fatigue and dyspnoea were included in the study. Those patients with preexisting lung disease were excluded from the study. Convenience sampling was done in this study.

The sample size was calculated using the following formula:


$$\begin{array}{ll} \text{n} & ={\text{Z}}^{2}\times \frac{\text{p}\times \text{q}}{{\text{e}}^{\text{2}}}\\ & =1.96^{2}\times \frac{0.50\times 0.50}{0.08^{2}}\\ & =150 \end{array}$$

Where,

n = minimum required sample size
Z = 1.96 at 95% Confidence Interval (CI)
p = prevalence taken as 50% for maximum sample size calculation
q = 1-p
e = margin of error, 8%

However, 153 samples were taken in this study.

Non-contrast chest CT scan were acquired in Hitachi— Scenaria Multidetector 128 slice CT scan using the helical mode volumetric High-resolution computed tomography (HRCT) with slice thickness of 1.0 mm and reconstruction interval of 0.6 mm using tube Voltage of 100-120 kilovoltage peak (kVp) and tube current of 100-500 mA.

Images were acquired in supine position in full inspiration. Multiplanar reconstruction of the images in axial, sagittal and coronal planes was done which were evaluated for the parenchymal changes in the lungs. Images were viewed on both lung windows (width: 1500 Hounsfield units (HU); level: -700 HU) and mediastinal windows (width: 350 HU; level: 40 HU) settings.

Chest CT images were evaluated by two radiologists. Lesions were evaluated based on the morphology as ground glass opacities, consolidation, fibrotic bands, septal thickening and additional findings like pleural effusion. Distribution of the lesion was divided into central and peripheral (defined by inner two third and outer one third of lung parenchyma) and anterior and posterior (defined by lung parenchyma anterior and posterior to the line drawn mid-way on axial CT images).^6^

The CT severity score (0-25) of the lung parenchymal involvement was calculated by the semi-quantitative CT scoring system. It is based on the extent of the lobar involvement of each 5 lung lobes lobar involvement (0 for 0%; 1 for <5%; 2 for 25-25%; 3 for 26-50%; 4 for 51-75%; 5 for >75%) and total score was calculated evaluating score in each lobe with score ranging from 0-25.^7,14^

Collected data were entered into the IBM SPSS Statistics 20.0 for statistical analysis. Point estimate and 95% CI were calculated.

## RESULTS

Out of 153 patients included in this study, abnormal chest CT findings were seen in 147 (96.07%) (92.9999.15, 95% CI). Among the patients with abnormal CT findings, the most common pattern of lung findings was pure ground-glass opacity (GGO) seen in 50 (34.01%) patients, only consolidation was seen in 12 (8.16%) patients. GGO with consolidations was seen in 78 (53.06%) patients. Diffuse GGO was seen in 7 (4.76%) patients. Apart from these features, patients also had other additional findings like septal thickening, crazy pavement, fibrotic band, and pleural effusion (Table 1).

**Table 1:** Abnormal chest CT findings and additional findings (n= 147).

Categories	Abnormalities	n (%)
Main finding	GGO with consolidation	78 (53.06)
	GGO	50 (34.01)
	Consolidation	12 (8.16)
	Diffuse GGO	7 (4.76)
Additional finding	Septal thickening	23 (15.64)
	Crazy pavement	3 (2.04)
	Fibrotic band	4 (2.72)
	Pleural effusion	7 (4.76)

Majority of the patients with abnormal chest CT 112 (76.19%) patients were found to have peripheral lung involvement and 35 (23.80%) patients had central and peripheral lung involvement. Majority of patients 122 (82.99%) had all lobes involved. Regarding distribution of parenchymal abnormalities, either both anterior and posterior lung area were involved, or only posterior area was involved (Table 2).

**Table 2:** Frequency of involvement of lung lobes, diseases localization, main pattern and additional findings (n = 147).

Categories	Findings	n (%)
Distribution	Peripheral and central	35 (23.80)
	Peripheral	112 (76.19)
Lung area	Anterior and posterior	79 (53.74)
	Posterior	68 (46.25)
Number of lobes involved	All	122 (82.99)
	One or more lobes	25 (17.00)
Lobar involvement	Right upper lobe	126 (85.71)
	Right middle lobe	124 (84.35)
	Right lower lobe	135 (91.83)
	Left upper lobe	129 (87.75)
	Left lower lobe	131 (89.11)

Majority of the patients with abnormal chest CT findings were males 107 (72.78%) and 64 (43.53%) patients were from the age group 26-50 years (Table 3).

**Table 3:** Demographic details (n = 147).

Characteristics	Findings	n (%)
**Sex**	Male	107 (72.78)
	Female	40 (27.21)
**Age (years)**	0-25	4 (2.72)
	26-50	64 (43.53)
	51-75	61 (41.49)
	>75	18 (12.24)

Using the CT score calculated by the semi-quantitative CT scoring system based on the extent of the lobar involvement of each five lung lobes, 68 (46.25%) patients showed moderate changes and 42 (28.57%) patients showed severe changes (Table 4).

**Table 4:** CT Severity among COVID-19 patients with abnormal chest CT findings (n = 147).

CT Severity	n (%)
Mild	37 (25.17)
Moderate	68 (46.25)
Severe	42 (28.57)

## DISCUSSION

The main pulmonary manifestation of the COVID-19 infection is COVID-19 pneumonia characterised by GGO and consolidations. Abnormal chest CT findings have been reported worldwide in COVID-19 patients. Most of the symptomatic COVID-19 patients in our study had findings in the chest CT (96.07%) which was comparable to other studies done in Italy,^15^ which reported findings in 96.83% of symptomatic COVID-19 patients and in China,^16^ which reported findings in 100% of symptomatic COVID-19 patients. In meta-analysis, the positive findings in symptomatic COVID-19 patient were 89.76%.^17^

The majority of patients in our study had bilateral peripheral GGO with or without consolidations. The distribution was predominantly peripheral with involvement of posterior areas of the lung in the majority of the patients. These findings were in concordance with various studies done in different countries.^6,7,13,15,18,19^ Other additional findings like septal thickening and crazy paving appearance were also seen in some of the cases in our study. Similar additional findings have been reported in COVID-19 patients in various studies done in other countries.^6,13,15-17^

Few symptomatic COVID-19 patients in our study showed associated pleural effusion (4.76%). In a review article based on various studies- the incidence of pleural effusion was 7.3% in COVID-19 patients and were seen commonly in critically ill patients.^20^ Most of the patients in our study showed moderate (46.25%) and severe (28.57%) severity score based on the COVID-19 CT severity index based on semiquantitative CT scoring system based on the extent of the lobar involvement of each 5-lung lobes.^7^

There were some limitations in this study. Large number of patients could not be included in the study. It was not feasible to perform CT scan on a large number of COVID-19 patients as the same CT scan machine had to be used for both COVID-19 and non-COVID-19 patients and the machine had to be disinfected everytime after performing CT scan of COVID-19 patients. CT scan was performed only once for a patient- so the course of lung parenchymal changes could not be evaluated.

## CONCLUSIONS

The prevalence of abnormal chest findings among symptomatic COVID-19 patients in our study was similar to the studies done in similar settings. Majority of the symptomatic COVID-19 patients showed abnormal chest CT scan findings in the form of GGO and GGO with consolidations. The knowledge of abnormal chest CT scan finding in symptomatic COVID-19 could contribute to accessing the severity of the disease and better planning the management in moderate to critically ill patients in designated COVID-19 hospitals.
